# Non-suicidal self-injury and other key predictors of suicide deaths among children and adolescents in England: a population-based case-control study

**DOI:** 10.1186/s12916-026-05170-7

**Published:** 2026-08-26

**Authors:** Ben Hoi-Ching Wong, David Odd, Karen Luyt, Vicky Sleap, Sylvia Stoianova, Tom Williams, Samantha Drew, Camilla Parker, Joan Asarnow, Dennis Ougrin

**Affiliations:** 1 East London NHS Foundation Trust, London, UK; 2 Centre for Psychiatry and Mental Health, Wolfson Institute of Population Health, Queen Mary University of London, London, UK; 3 National Child Mortality Database, University of Bristol, Bristol, UK; 4 Great Ormond Street Hospital for Children NHS Foundation Trust, London, UK; 5 South London and Maudsley NHS Foundation Trust, London, UK; 6 University of California, Los Angeles, USA; 7 Institute of Mental Health, Ukrainian Catholic University, Lviv, Ukraine

**Keywords:** Suicide, Non-suicidal self-injury, Self-harm, Epidemiology, Risk factors, Child, Adolescents

## Abstract

**Background:**

Suicide is a leading cause of mortality in young people globally. A history of prior self-harm, with or without suicidal intent, is associated with increased risks of future suicide attempts (SAs) or suicides. While non-suicidal self-injury (NSSI) is likely a predictor of SA, its particular association to suicide deaths remains unexplored.

**Methods:**

This is a population-based case-control study in England. Cases were all individuals who died by suicide before their 18th birthday (*n* = 240), between April 2019 and March 2021. Controls (*n* = 240) were age- and sex-matched individuals who died from other unexpected causes. Conditional logistic regression was used to estimate odds ratios for self-harm history and other risk factors, adjusting for ethnicity and socioeconomic deprivation. A fully adjusted multivariable model was formed to estimate associations between each risk factor and suicide death, adjusting for all measured factors.

**Results:**

A history of NSSI was strongly associated with subsequent suicide death (partially adjusted OR, 27.8; 95% CI, 9.5–81.0; fully adjusted OR, 9.2; 95% CI, 2.1–39.5). The association remained significant in sensitivity analyses restricted to those with only NSSI history without SA (partially adjusted OR, 7.7; 95% CI, 3.6–16.5). History of SA was also significant (adjusted OR, 64.2; 95% CI, 8.8–468; fully adjusted OR, 12.3; 95% CI, 0.4–357). Notably, 42.6% of suicides occurred in individuals without previous self-harm, and 71% of suicides occurred on the first documented act of intentional self-injury or self-poisoning. MDMA was recorded in 14 suicide cases, and alcohol use in 34 suicide cases, regardless of whether the intake resulted in overdose. Fully adjusted model found that known NSSI history, known suicidal ideation history, and bullying victimization remained independently associated with suicide death.

**Conclusions:**

NSSI and SA are both major predictors for suicide in young people. Risk assessments should aim to include all significant factors. These findings highlight the need for holistic clinical screening for all forms of self-harm regardless of intent, indicated suicide prevention intervention for those identified, and enhanced access to mental health services.

**Supplementary Information:**

The online version contains supplementary material available at https://doi.org/10.1186/s12916-026-05170-7.

## Background

Suicide is the third leading cause of death for young people aged 15–29 worldwide [1], with a mortality rate estimated at 3.8 per 100,000 among 10-19-year-olds [2]. Self-harm, defined as intentional self-poisoning or self-injury regardless of motive [3], is one of the strongest predictors of suicide [4, 5]. Increases in youth suicide deaths and nonfatal self-harm have significantly increased globally in recent years [1, 6], raising concern regarding the potential for increasing suicide death rates.

Self-harm can be broadly categorised based on the reported intent of the act, into suicide attempts (SAs), defined as self-harm with suicidal intent, and non-suicidal self-injury (NSSI), self-harm without explicit evidence of suicidal intent [7–9]. Distinguishing between these two different types of self-harm can be challenging, however, as youths often find it difficult to accurately describe their intent and some self-harm acts have unclear intent [9]. The UK National Institute for Health and Care Excellence (NICE) guidelines recommend steering away from an intent-based categorisation [3]. as it overlooks that intent could be fluctuating or ambivalent. Stratification of risk could be misleading and potentially harmful by resulting in inaccurate care communication and assessment. In practice, however, the questions of suicidal intent often follow questions of self-harm and recorded together [10]. Internationally, NSSI is also a widely used label adopted in the DSM-5 [11]. Research has identified distinction between NSSI and SA across epidemiological patterns [4], clinical profiles [12], risk factors [7, 13], genetic architectures [14], and treatment responses [15]. Although some youths engage in both NSSI and SA, their stated functions are distinctly different. NSSI is often described as a temporary escape from distress, whereas SAs are associated with a desire to die, and often a permanent end to consciousness and psychological pain [16]. Nevertheless, there are clear associations across these two types of self-harm. NSSI is linked to a higher risk of subsequent SA [17–19]. The risk of SA potentially increases with longer NSSI history, as adolescents with NSSI histories have been found to have a higher emotional reactivity to stress and reduced distress tolerance [20].

To date, while research supports associations between NSSI and non-fatal SAs [19], and between suicide death and self-harm disregarding intent (defined to include both SAs and NSSI) [5], the direct association between NSSI specifically and suicide deaths remains to be evaluated [21, 22]. This link may not be directly extrapolated because those who engaged in non-fatal SA and those who died by suicide may be distinct albeit overlapping populations [23]. Both the individual and compounding effects of NSSI and SA on increased risks for adverse clinical outcomes are well-documented in previous research [24]. However, it is unclear if NSSI alone remains a strong risk factor for suicide mortality specifically [5, 18].

This study aimed to investigate this gap in the literature. We aimed to improve our understanding of the predictors of suicide death, using a unique national dataset of all deaths of children and adolescents in England.

## Methods

### Design and setting

This is a population-based case-control study comparing suicide deaths with control deaths of other causes (e.g. trauma, unexpected medical conditions). We utilised statutory data reported to the National Child Mortality Database (NCMD). In England, a mandatory child death review (CDR) process follows any child’s death (aged under 18) [25]. Comprehensive information about the child and the death is gathered systematically through semi-structured interviews and linked routine records, combining inputs from the family, peers, coroner, police, and all professionals who had cared for the child (e.g. education, primary care, social care), then reviewed by the multi-agency Child Death Overview Panels (CDOPs).

## Study population

The cases of the current study were children and adolescents who were usual residents in England and died by suicide before their 18th birthday, between 01 April 2019 and 31 March 2021. The controls were children and adolescents who died of other unexpected causes in England, individually matched to cases by age and sex at a 1:1 ratio, both known confounders of suicide and self-harm. According to statutory guidance, unexpected deaths are ‘a descriptive term used at the point of presentation for the death of an infant or child whose death was not anticipated as a significant possibility 24 hours before the death, or where there was a similarly unexpected collapse leading to or precipitating the events which led to the death’ [25]. This was selected to be the control because suspected suicides and unexpected deaths (categorised at point of death before investigation) are subject to equivalent investigation and data collection processes, minimising potential information biases. Conversely, for instance, psychiatric history might not have been asked for expected deaths from chronic medical conditions. Informants around deaths are also more likely and motivated to provide comprehensive information about the child [26].

### Sampling

Cases were identified via a two-step process to reduce misclassification. Each extracted potential suicide death within the inclusion timeframe was comprehensively reviewed by the lead author (a clinical academic) and rated on whether it was a probable suicide (probable/improbable/undetermined), based on all available evidence, e.g. coroner reports, interview transcripts of stakeholders, records of the public mental healthcare providers (CAMHS; Child and Adolescent Mental Health Services) and general practitioners. This additional assessment followed methodology commonly adopted in suicide research [27, 28], classifying self-inflicted deaths with equivocal suicidal motivation as probable suicides (e.g. drowning from a jump into water after an adverse event), and self-inflicted deaths with no suspicion of suicidal thinking as improbable (e.g. overdose from a recreational drug at a party with no mental health history/concern). All probable suicides along with any undetermined deaths that were originally categorised as suicide by CDOP, were included as cases. Deaths caused by deliberate ingestion of substances were excluded if there was sufficient evidence that it was not intended to cause harm/death (based on witness statements, typical recreational or therapeutic levels, patterns of use, and mental health history).

Controls matching the same sex and age of death to suicide cases were identified if there was a joint-agency response or the suspected category of death assigned at the point of 48-hour notification by three independent clinicians was ‘Trauma’ or ‘Sudden Unexpected Death in Infancy or Childhood’, as per NCMD methodology. The eligible control death that occurred the closest in time to each suicide case was selected.

### Measures

Measure coding was performed by lead author. Although data sensitivity and capacity restrictions limited measure coding to one clinical academic, reliability of the data were established through five mechanisms: (1) source data were collected and verified by designated child review partners via standardised statutory protocols; (2) variables and coding framework for this study were specified a priori based on existing theoretical and empirical evidence; (3) all recorded data, and the lack thereof, were triangulated across available documents and records of each child to verify accuracy; (4) intra-rater reliability was assessed and ensured at regular intervals (every 50 subjects; κ > 0.8); and (5) any coding uncertainties were resolved through consensus with the research team comprising of data processors, epidemiologists, and child suicide experts.

Four sociodemographic confounders [5, 18] were recorded: age, sex, socioeconomic deprivation, and ethnicity (White British/Black or Black British/Asian or Asian British/Other White/Other). Socioeconomic deprivation was measured using the official Index of Multiple Deprivation (IMD) of the child’s permanent residence, IMD is an official UK government measure that ranks small neighbourhoods by levels of socioeconomic disadvantage combining domains such as, income, education, crime, living environment, etc [29]. Additional coding was undertaken to contextualise the suicides: whether the suicide plan was communicated to others before the act, the choice of substance used in overdose/self-poisoning, and whether/how social media contributed to the suicide.

The primary factor of interest was a history of NSSI, defined as all forms of deliberate injury to own body tissues where the intention (disclosed by the individual) is not to kill oneself [30], including potential internal tissue damage such as overdose or self-poisoning [8], which are frequently reported to be non-suicidal [31, 32]. We included self-harm that was potentially fatal but reported as non-suicidal (e.g. overdose) and excluded non-fatal self-harm with unknown intent.

For secondary factors, we included four categories of potential risk factors based on existing literature on suicide [4, 18, 33]. Further coding was undertaken by the lead author after reviewing all available information collected during CDR, including text fields, predefined categorical selections, uploaded documents, and reports. Psychiatric factors included history of suicidal ideation, history of SA, and selective serotonin reuptake inhibitors (SSRI) prescription. Suicidal ideation history was defined as any known thought or plan about ending one’s own life, regardless of whether it was acted upon. SA is any intentional attempt to end one’s own life regardless of the method, without resulting in suicide death.

Precipitating factors included whether there was any recent NSSI in the last 24 h of life, excluding the act that led to eventual death, and usage of illicit drugs or alcohol before death, regardless of whether the intake resulted in overdose. This includes any substance that likely influenced the child’s judgment before death, based on toxicology reports or witness accounts.

Psychosocial factors included family history of any self-harm, exposure to any self-harm in non-family members, intact family structure, whether the child was victim of bullying, and exposure to domestic/sexual abuse. Family history encapsulates any NSSI, SA and suicide death by family members of the child, whereas non-family exposure included any such act by non-family members that was known to the child. Intact family structure was defined as the child living with both biological parents before death.

Service history included CAMHS involvement history, any barrier to accessing CAMHS (as identified in the investigative process), and ongoing social care involvement. CAMHS involvement included any attended, clinically relevant appointments such as psychosocial assessments and/or therapeutic sessions, excluding open waitlist cases. Barriers were also recorded qualitatively, including any CDOP-identified modifiable factor that prevented the child from receiving appropriate CAMHS support.

All variables were recorded as binary outcomes and coded as missing when it was directly stated as ‘unknown’, or when the relevant questions were not completed in the CDR process.

### Statistical analysis

Summary tables were produced to summarise the sociodemographic characteristics and suicide characteristics. All analyses were conducted using Stata/MP 18.0. Missing data on predictor variables were imputed using multivariable imputation with chained equations (MICE), with the -mi impute chained- command (see eAppendix). The imputation model included all potential risk factors, the unmatched confounders (ethnicity and deprivation), the strata variable (identifying matched pairs), and the outcome variable (case/control). Ten imputed datasets were created. We assumed data were missing at random, conditional only on observed variables. Additional details of the imputation model and missingness are summarised in Additional file 1: Tables S1-S3. Estimates from the multiply imputed datasets were pooled using Rubin’s rules.

To address our primary research question of whether NSSI history and/or SA history were associated with suicide death, separate conditional logistic regression models were fitted for NSSI history and SA history, respectively, forming a stratum for each matched pair in each model. Known confounders were adjusted through the matching design (age and sex) and as covariates in the partially adjusted regression models (ethnicity and socioeconomic deprivation). For sensitivity analyses, we also inspected the individual and combined impacts of NSSI and SA. Non-overlapping self-harm history variables were recoded and tested: (i) NSSI-only history, (ii) SA-only history, and (iii) NSSI + SA histories. Conditional logistic regression was repeated in sensitivity analyses, and for all other collected risk factors to assess their standalone relationships with suicide. Adjusted OR for each exposure was calculated, indicating their clinically relevant association to suicide, adjusted for ethnicity and socioeconomic deprivation.

To inspect the effects of the variables after adjusting for other covariates, all factors associated with suicide at *p* < .10 in their respective partially adjusted models were further entered simultaneously into a fully adjusted conditional logistic regression model, along with the two un-matched confounders (ethnicity and socioeconomic deprivation). All candidate variables were checked for multicollinearity by assessing variance inflation factors (VIF), with a high value (> 5) leading to the variable being excluded to prevent unstable estimates.

### Patient and public involvement

This study is co-authored by a lived experience parent and child psychiatry clinicians who contributed to the interpretation of results. This research responds to concerns of the families of children and adolescents who died of suicide. The findings will be disseminated to clinical services and patient communities.

## Results

In total, 240 suicide or probable suicide cases were recorded in England between April 2019 and March 2021. The majority were boys (65.0%) and White British (72.1%); with a mean age of 15.5 years (standard deviation = 1.8 years). The sociodemographic characteristics of suicide cases and controls are summarised in Table 1.

**Table 1:** Sociodemographic characteristics of the sample

	Suicide cases	Controls^a^
	*n* (%)	*n* (%)
**Total**	*N* = 240	*N* = 240
**Age**,** years**		
Mean (SD)	15.5 (1.8)	15.5 (1.8)
**Sex**		
Male	156 (65.0%)	156 (65.0%)
Female	84 (35.0%)	84 (35.0%)
**Difference in dates of death between each matched case-control pair**,** days**		
Mean (SD)	44.3 (126.7)	44.3 (126.7)
**Ethnicity**		
White British	173 (72.1%)	141 (58.8%)
Black or Black British	13 (5.4%)	40 (16.7%)
Asian or Asian British	22 (9.2%)	37 (15.4%)
White - Other	23 (9.6%)	16 (6.7%)
Other ethnicity groups	7 (2.9%)	4 (1.7%)
Not known	2 (0.8%)	2 (0.8%)
**Index of Multiple Deprivation (IMD)** ^**b**^		
1^st^ decile	30 (12.5%)	35 (14.6%)
2^nd^ decile	27 (11.2%)	37 (15.4%)
3^rd^ decile	20 (8.3%)	38 (15.8%)
4^th^ decile	29 (12.1%)	29 (12.1%)
5^th^ decile	23 (9.6%)	26 (10.8%)
6^th^ decile	22 (9.2%)	18 (7.5%)
7^th^ decile	25 (10.4%)	15 (6.2%)
8^th^ decile	24 (10.0%)	19 (7.9%)
9^th^ decile	24 (10.0%)	9 (3.8%)
10^th^decile	15 (6.2%)	13 (5.4%)
Not known	1 (0.4%)	1 (0.4%)

^a^ Controls were unexpected deaths of other unexpected causes (see Methods); ^b^ Official data published by the Ministry of Housing based on postcode of residence, 1^st^ decile is the most deprived and 10^th^ decile is the least deprived. Abbreviations: SD, standard deviation

In 193 (80.4%) of recorded suicides, there was no evidence that an explicit suicide plan was communicated to others before the act (Table 2). Hanging/self-strangulation was the most common method of suicide (65.0%), followed by jumping/lying before a train and deliberate overdose/self-poisoning (both at 11.2%). MDMA (3,4-methylenedioxymethamphetamine) was the most common substance contributing to overdose suicides, accounting for nearly a third of overdoses. MDMA was also the second most commonly consumed substance prior to death in all suicides (5.8%; Table 3), while alcohol consumption was detected in one in seven suicides (14.2%), compared with one in twenty control deaths (5.0%).

**Table 2:** Characteristics of suicide deaths (*N* = 240)

	Suicide cases
	*n* (%)
Communicated plan before suicide	
Yes	41 (17.1%)
No	193 (80.4%)
Not known	6 (2.5%)
**Method**	
Hanging/self-strangulation	156 (65.0%)
Deliberate overdose/self-poisoning	27 (11.2%)
MDMA	8 (3.3%)
Other substances (see footnote)	20 (8.3%)
Not known	1 (0.4%)
Jumping/lying before train	27 (11.2%)
Jumping from height	18 (7.5%)
Other methods (including suffocation/asphyxiation, drowning, firearm, inhalation of aerosol, walking into road traffic)	12 (5.0%)
**Social media involved**	
Yes	27 (11.2%)
As a trigger (of distress)	21 (8.8%)
To communicate suicide plan, or using suicide forums	6 (2.5%)
No	213 (88.8%)

Note: Cells with small numbers (*n* < 5) are combined to avoid identification of individuals. The substances listed in this table may overlap, as some individuals consumed more than one substance before death. Other substances in overdose suicides include: morphine, propranolol, cyclizine, sodium nitrate, cocaine, heroin, alcohol, aspirin, paracetamol, flubromazolam, metformin, sodium azide, oxycodone, pregabalin, citalopram. Abbreviations: MDMA, 3,4-Methylenedioxymethamphetamine (Ecstasy)

**Table 3:** Substance usage (regardless of overdose) before death

	Suicide cases
	*n* (%)
**Alcohol**	34 (14.2%)
**MDMA**	14 (5.8%)
**Cocaine**	8 (3.3%)
**Marijuana**	7 (2.9%)
**Heroin** ^a^	< 5
**LSD** ^a^	< 5
**Ketamine** ^a^	< 5
	**Controls**
	**n (%)**
**Alcohol**	12 (5.0%)
**Marijuana**	13 (5.4%)
**MDMA** ^a^	< 5
**Cocaine** ^a^	< 5

^a^ Cells with small numbers (*n* < 5) are suppressed to avoid identification of individuals. The substances listed in this table may overlap, as some individuals consumed more than one substance before death. Abbreviations: MDMA, 3,4-Methylenedioxymethamphetamine (Ecstasy); LSD, Lysergic acid diethylamide

More than 40% of suicide deaths had no lifetime history of any self-harm irrespective of suicidal intent (Table 4). Altogether, 70.9% of the suicide cases had died on their first known attempt on life. Amongst those who had self-harmed before (NSSI and/or SA), about half (*n* = 65; 50.9%) had histories of only NSSI with no recorded history of SA. Laceration/self-cutting was the most common method (43.9%) of the last recorded self-harm, regardless of intent, before death, in both cases and controls.

**Table 4:** Summary of self-harm history

	Suicide cases	Controls
	*n* (%)	*n* (%)
**Any history of self-harm**	*N* = 230	*N* = 240
Had histories of both SA and NSSI	52/230 (22.6%)	< 5
History of SA only	15/230 (6.5%)	< 5
History of NSSI only	65/230 (28.3%)	12/240 (5.0%)
Never engaged in NSSI nor SA	98/230 (42.6%)	225/240 (93.8%)
No known history of NSSI, SA, or suicidal ideation	62/230 (27.0%)	222/240 (92.5%)
**Last known self-harm method before death**	*N* = 132	*N* = 15
Laceration/skin damage	58/132 (43.9%)	9/15 (60.0%)
Deliberate overdose/self-poisoning	22/132 (16.7%)	< 5
Hanging/self-strangulation	17/132 (12.9%)	< 5
Head banging	7/132 (5.3%)	0
Other methods (including walking into road traffic, punching wall, jumping into water, alcohol intoxication, jumping from height)	9/132 (6.8%)	4/15 (26.7%)
Method not recorded	19/132 (14.4%)	0

Note: Cells with small numbers (*n* < 5) are combined or suppressed to avoid identification of individuals. Percentages are calculated based on the applicable sample with available/applicable data (N). Self-harm history was missing for 10 suicide cases. Abbreviations: SA, suicide attempt; NSSI, non-suicidal self-injury

Table 5 presents the results of conditional logistic regression. Adjusting for ethnicity and socioeconomic deprivation, children and adolescents with a history of NSSI had 27.8 times the odds of dying by suicide compared to those without (95% CI, 9.5–81.0), whilst youths with a history of SA had 64.2 times the odds of dying by suicide compared to those without (95% CI, 8.8–468). Sensitivity analyses using mutually exclusive self-harm categories tested the theoretical individual and combined impacts of NSSI and SA. NSSI-only history was associated with 7.7-fold increase in suicide death odds (95% CI, 3.6–16.5). Having a self-harm history of both NSSI and SA was found to increase odds of suicide death by 26.1 times (95% CI, 6.2–109). Odds ratio for SA-only history could not be reliably estimated due to sparse data.

**Table 5:** Odds ratios (95% CI) for the association between exposure variables and suicide death

Exposure variables	Suicide cases		Controls		Conditional logistic regression		
	*N*	*n* (%)	*N*	*n* (%)	Crude OR (95% CI)	Partially adjusted^a^ OR (95% CI)	Fully adjusted^b^ OR (95% CI)
Self-harm history							
NSSI history	230	117 (50.9%)	240	14 (5.8%)	28.4*** (10.1–79.8)	27.8*** (9.5–81.0)	9.2** (2.1–39.5)
SA history	231	67 (29.0%)	240	< 5	65.7*** (9.1–473)	64.2*** (8.8–468)	12.3 (0.4–357)
***Sensitivity analyses***							
NSSI-only history	230	65 (28.3%)	240	12 (5.0%)	8.1*** (3.8–17.1)	7.7*** (3.6–16.5)	
SA-only history^c^	230	15 (6.5%)	240	< 5	n/a	n/a	
NSSI + SA histories	230	52 (22.6%)	240	< 5	26.3*** (6.4–108)	26.1*** (6.2–109)	
**Secondary factors**							
*Psychiatric factors*							
Suicidal ideation history	231	150 (64.9%)	240	10 (4.2%)	29.6*** (12.1–72.1)	29.8*** (11.9–75.2)	20.4*** (5.2–80.8)
SSRI prescription	240	29 (12.1%)	240	< 5	9.7*** (3.0-31.7)	9.5*** (2.8–32.0)	1.3 (0.1–18.0)
*Precipitating factors*							
NSSI within last 24 h before death^c^	231	10 (4.3%)	240	0	n/a	n/a	
Illicit drug use before death	229	24 (10.5%)	236	15 (6.4%)	1.6 (0.8–3.2)	1.7 (0.8–3.6)	
Alcohol use before death	226	34 (15.0%)	237	12 (5.1%)	3.5*** (1.7–7.4)	3.4** (1.6–7.3)	1.8 (0.5–6.7)
*Psychosocial factors*							
Family history of self-harm	233	51 (21.9%)	223	14 (6.3%)	3.4*** (1.8–6.5)	3.6*** (1.8–7.1)	2.0 (0.5–6.9)
Exposure to self-harm in non-family	240	25 (10.4%)	240	< 5	25.0** (3.4–185)	38.9*** (4.8–316)	27.7 (0.3–2290)
Intact family structure	235	100 (42.6%)	235	108 (46.0%)	0.9 (0.6–1.2)	0.8 (0.5–1.2)	
Bullying victimisation	240	16 (6.7%)	240	< 5	16.0** (2.1–121)	16.14** (2.1–127)	202** (3.6-11371)
Exposure to abuse	239	55 (23.0%)	240	35 (14.6%)	1.9* (1.1–3.1)	2.03* (1.2–3.5)	1.4 (0.5–3.9)
*Service history*							
CAMHS involvement (current/historic)	240	95 (39.6%)	239	30 (12.6%)	4.4*** (2.7–7.2)	4.8*** (2.8–8.3)	0.79 (0.2–2.6)
Barrier identified in accessing CAMHS	240	73 (30.4%)	240	23 (9.6%)	3.9*** (2.3–6.7)	3.9*** (2.2–6.9)	0.32 (0.1-1.0)
Active social care involvement	240	23 (9.6%)	240	27 (11.3%)	0.8 (0.5–1.5)	1.0 (0.5–1.9)	

Note: Cells with small numbers (*n* < 5) are combined or suppressed to avoid identification of individuals. Regression analyses were conducted with multiply imputed datasets. *p < .05; **p ≤ .01; ***p ≤.001. Abbreviations: OR, odds ratio; CI, confidence interval; NSSI, non-suicidal self-injury; SA, suicidal attempt; CAMHS, Child and Adolescent Mental Health Service

^a^Partially adjusted model adjusted for ethnicity and socioeconomic deprivation

^b^Fully adjusted model included ethnicity, socioeconomic deprivation, and all variables with *p* < .1 in the partial adjusted models, entered simultaneously

^c^Analysis not possible due to insufficient cell count for matched comparison

Eleven additional variables were found with significant individual effects when ethnicity and socioeconomic deprivation were accounted for. A recorded history of suicidal ideation (partially adjusted OR, 29.8; 95% CI, 11.9–75.2) was significantly associated with suicide death. Children and adolescents who died by suicide had higher odds of an SSRI prescription, to have consumed alcohol before death, and to have a family member or non-family member who had any form of self-harm history. Experience of bullying or abuse was linked to higher rates of suicide compared to other causes of death. CAMHS involvement was more prevalent among suicide cases. However, barriers to accessing CAMHS were also more frequently identified (See Additional file: Table S4 for primary barriers identified).

All covariates identified above with evidence of a likely association (*p* < .10) after adjusting for confounders were entered into a fully adjusted model. NSSI history (fully adjusted OR, 9.2; 95% CI, 2.1–39.5), suicidal ideation history (fully adjusted OR, 20.4; 95% CI, 5.2–80.8), and bullying victimisation (fully adjusted OR, 202; 95% CI, 3.6-11371) remained the independently significant factors. SA history was found to have a large but non-significant association with suicide death. Low counts of exposed controls and extreme confidence intervals for both bully victimisation and SA history likely reflected unstable estimates limited by sparse data.

## Discussion

This national study comprehensively reviewed high-quality mortality data collected through a combination of routine records and standardised interviews. Our findings are, to our knowledge, the first direct evidence that NSSI is a significant risk factor for suicide death. Thirteen variables were found with significant adjusted odds ratios after accounting for sociodemographic confounders. These variables indicate elevated suicide risk among youths. Clinicians should proactively assess for histories of NSSI, SA, and suicidal ideation, alongside other factors such as exposure to self-harm in any non-family members. The fully adjusted model suggested that known histories of NSSI, suicidal ideation, and bullying victimisation are associated with suicide death independently after controlling for all associated risk factors. Suicidal ideation history was found to have a stronger effect than NSSI history. A low rate of SA history in controls limited statistical power to investigate its effect.

In previous UK samples, a history of self-harm (disregarding intent) was a risk factor alongside bullying and other life adversities [34], and was found in approximately half of young people who died by suicide [35]. The present study adds to literature by supporting the effects of NSSI specifically. The association between NSSI history and suicide death remained robust in sensitivity analysis excluding co-occurring suicide attempts, and after accounting for all other covariates in the fully adjusted model, demonstrating its theoretical significance in addition to its clinical relevance. A history of SA was also strongly associated to suicide death, consistent with prior research [5, 36]. It had the largest adjusted odds ratio, estimated to increase suicide odds by 64.2 times. However, its association with suicide was attenuated and non-significant in the fully adjusted model. Beyond limitations from sparse data, this could reflect confounding by other covariates and/or other underlying mechanisms such as mediation. Distinguishing between these interpretations would require larger samples and a dedicated study design. Previous literature has well-documented that NSSI increases further risks of SA [18]. As we found an increased effect of NSSI + SA on suicide death, NSSI may serve as a critical early warning sign before suicide risk compounds. It remains to be studied whether youths who engage in both NSSI and SA differentiate from those who only ever engage either. A recorded history of suicidal ideation helps further identify suicide risk, as it was a substantial predictor and remained independently significant in the fully adjusted model. While the research continues to develop, we now have rigorous RCTs that provide support for the efficacy of intervention approaches for reducing NSSI, SA, and SI in adolescents, with the strongest available cross-study data supporting the benefits of dialectical behaviour therapy (DBT) and DBT-informed approaches [37–39].

Nevertheless, 27% of suicide cases had no documented history of NSSI, SA, or suicidal ideation. Instead of relying on these histories as the only warning signs, clinicians and researchers must also acknowledge other risk factors, as certain sub-groups of youths may appear well-adjusted before death. A large proportion of the children and adolescents in the sample of suicide decedents died on their first recorded attempt. About 70% of suicide cases had no documented history of SA, and more than 40% had no documented history of any prior self-harm regardless of intent, matching figures from a previous American cohort [40]. NSSI, albeit more prevalent, was only present in about half of the youths who died by suicide. These findings are comparable to previous data in England [34]. It is unclear if this reflects that children and adolescents underestimate the fatality of their self-harm, or that they have the knowledge of effective, fatal methods of suicide. To minimise suicide deaths, prevention strategies must consider the possibility that findings from adolescents who made non-fatal attempts may not necessarily generalise to those at risk of completing suicide on their first attempt.

Variables significant when partially adjusted but insignificant in the fully adjusted models potentially represented overlapping subgroups. Even though their associations were not independent of other measured risk factors, they remain clinically important and should be used to construct a broader risk profile in risk assessment. This included alcohol consumption, family history of self-harm, and exposure to self-harm in others, aligning with existing evidence [4, 34]. CAMHS involvement history and barriers in accessing CAMHS being significant predictors highlight the need for better containment of suicide risks in young people who have had contact with CAMHS and addressing access barriers, potentially through better collaboration with third-sector mental health providers.

Presence of illicit drug use did not have a direct association with suicide. However, MDMA consumption was particularly common in suicides regardless of overdose, supporting previous research [41]. The overall variety of recreational substances found in overdoses distinctly juxtaposes with past reports where paracetamol was the predominant substance of choice [42]. Our observation reinforces the short-term suicide risk associated with illicit substance usage [5], particularly in adolescents with suicidal ideation or NSSI history [18]. Targeted public health and harm reduction strategies are needed for high-risk groups.

This study is subject to at least five limitations. First, it is unknown whether all data were collected with equal robustness. Our findings assume all relevant information would have been gathered since the statutory joint agency response include comprehensive proformas, data linkage, and detailed interviews. Although all included deaths, regardless of causes, underwent equivalent information-gathering procedures, interviewer biases might be introduced based on the circumstances around a death. Mental health related exposures might be less likely to be identified in certain control populations (e.g. traffic incidents as passenger), inflating the associations observed. Second, the data were retrospective and could not establish causal relationships. Any associations and inferred theoretical pathways would need to be tested in dedicated research. Third, there was insufficient information to ascertain the temporal relationship between multiple self-harm acts to investigate whether there was any linear elevation or method switching in self-harm [5]. Fourth, due to limited funding and capacity, only one researcher was involved in coding, therefore inter-rater reliability could not be measured, although a strong intra-rater reliability was ensured. Fifth, our data could not capture unobserved or undisclosed self-harm and suicidal ideation, as well as some other potentially relevant variables, including gender, sexuality, and any potential long-term impacts of the coronavirus pandemic. Our data do, however, include a year before and after the onset of the outbreak (Additional file 1: Fig. S1).

## Conclusions

NSSI in children and adolescents is a strong indicator of risk of suicide death and must be targeted in any suicide prevention strategies. Young people who engage with any self-harm (NSSI and/or SA) represent vulnerable groups with heightened odds of suicide death. The fully adjusted model identified NSSI history, suicidal ideation history, and bullying victimisation as independent predictors of suicide death in our data, although ideally clinicians should monitor all variables with significant adjusted odds ratios as they indicate elevated suicide risk. Consumption of MDMA has an unclear but potentially important role and should be closely monitored. Overall, study results underscore the heterogeneity in pathways to suicide deaths, with over half of youths dying on their first suicide attempt with no evidence of prior self-harm behaviour. A comprehensive assessment is needed for each at-risk young person, considering potential risks and protective processes that can be mobilised to prevent death or morbidity, even in the absence of any apparent self-harm behaviour, as the first attempt could be fatal. Indeed, interventions that have shown promise in reducing suicidal and self-harm behaviour have frequently emphasised case conceptualisations and individual tailoring to address the particular individual and socio-ecological risk and protective factors for each individual youth [37, 38]. Policy makers should focus resources on increasing access to these interventions for young people with NSSI in order to minimise potential suicide.

## Supplementary information

Below is the link to the electronic supplementary material.

Supplementary Material 1: Additional File 1: Tables S1-S4. Fig. S1.

## Data Availability

Aggregate data supporting the study’s findings are available on request subject to approvals by HQIP. The full data is not publicly available due to containing sensitive information that could compromise the privacy of individuals.

## Abbreviations

CAMHS: Child and Adolescent Mental Health Services
CDOP: Child Death Overview Panel
CDR: Child Death Review
IMD: Index of Multiple Deprivation
MDMA: 3,4-methylenedioxymethamphetamine
MICE: Multivariable imputation with chained equations
NCMD: National Child Mortality Database
NSSI: Non-suicidal self-injury
SA: Suicide attempt
SSRI: Selective serotonin reuptake inhibitors
VIF: Variance inflation factors
