# Supplementary material for: Non-suicidal self-injury and other key predictors of suicide deaths among children and adolescents in England: a population-based case-control study
Source: BMC Med. 2026 Aug 26;24:521. doi: 10.1186/s12916-026-05170-7 (PMC13615632; doi:10.1186/s12916-026-05170-7)
Supplement: Supplementary file 1 [file 12916_2026_5170_MOESM1_ESM.docx]

**Supplementary materials**

**Multiple imputation model**

Missingness was examined to determine the variables to be imputed: suicidal ideation history, suicide attempt history, alcohol use before death, NSSH in the last 24 hours before death, family history of self-harm, CAMHS involvement, exposure to abuse, illicit drug use before death, IMD, ethnicity.

To maximise accuracy of imputation, all other variables used in the formal analyses were entered into the model: outcome variable, strata variable, depressive disorder diagnosis, anxiety disorder diagnosis, bipolar disorder diagnosis, psychotic disorder diagnosis, eating disorder diagnosis, substance misuse disorder diagnosis, ASD diagnosis, ADHD diagnosis, victim of bullying, exposure to self-harm in non-family members, active social care involvement, SSRI prescription, any barrier to accessing CAMHS.

Multivariate imputation with chained equations (MICE) was adopted incorporating the -augment- option to address potential perfect prediction. Ordered logistic regression was used for IMD because of the ordinal nature of decile index. Multinomial logistic regression was used for ethnicity. Logistic regression was used for all other variables because of binary coding.

Details of missingness are summarised in Tables S1-S3 below.

**Table S1. Distribution of missingness**

|  | **Suicide cases** | | **Controls** | |
| --- | --- | --- | --- | --- |
|  | **Frequency** | **Percentage** | **Frequency** | **Percentage** |
| **Individuals with missing data** | 29 | 12.08% | 28 | 11.67% |
| **Variable with missing data** |  |  |  |  |
| NSSH history | 10 | 4.17% | 0 | 0 |
| Suicidal attempt history | 9 | 3.75% | 0 | 0 |
| Suicide ideation history | 9 | 3.75% | 0 | 0 |
| NSSH within last 24 hours before death | 9 | 3.75% | 0 | 0 |
| Illicit drug use before death | 11 | 4.58% | 4 | 1.67% |
| Alcohol use before death | 14 | 5.83% | 3 | 1.25% |
| Family history of self-harm | 7 | 2.92% | 17 | 7.08% |
| Intact family structure | 5 | 2.08% | 5 | 2.08% |
| Exposure to abuse | 1 | 0.42% | 0 | 0 |
| Any CAMHS involvement | 0 | 0 | 1 | 0.42% |
| Ethnicity | 2 | 0.83% | 2 | 0.83% |
| Deprivation (IMD) | 1 | 0.42% | 1 | 0.42% |

**Table S2. Pattern of missing data**

**Table S3. Prediction of complete record**

Table S3 describes the odds of having a complete record. A summary variable indicating whether the death has complete data was regressed on each sociodemographic variable and outcome indicator.

| **Variable** | **Odds ratio (95% confidence interval)** |
| --- | --- |
| Sex  Male  Female | 1 (base)  0.630 (0.338, 1.174) |
| Age of death  (for every 1-year increment) | 0.993 (0.849, 1.163) |
| Ethnicity  White British  Black British  Asian British  Other White | 1 (base)  0.651 (0.221, 1.913)  1.627 (0.757, 3.498)  0.911 (0.306, 2.717) |
| Deprivation  (for every decile increment on IMD) | 0.834 (0.744, 0.935) |
| Outcome  Non-suicide (control)  Suicide (case) | 1 (base)  1.041 (0.598, 1.809) |

**Table S4. Primary identified barrier to accessing CAMHS**

|  | **Suicide cases**  **n (%)** | **Controls**  **n (%)** |
| --- | --- | --- |
| Patient non-engagement | 34 (14.2%) | 15 (6.3%) |
| Not meeting threshold | 9 (3.8%) | 3 (1.3%) |
| No referral made | 9 (3.8%) | 1 (0.4%) |
| Delay in appointment | 5 (2.0%) | 0 |
| Lack of clarity of referral pathway | 3 (1.3%) | 2 (0.8%) |
| Failure to address needs | 3 (1.3%) | 0 |
| Lockdown/self-isolation | 2 (0.8%) | 2 (0.8%) |
| Triage difficulties | 2 (0.8%) | 0 |
| Instability of home environment | 1 (0.4%) | 0 |
| Insufficient information on referral | 1 (0.4%) | 0 |
| Declined as receiving private mental health care | 1 (0.4%) | 0 |
| Geographical constraint | 1 (0.4%) | 0 |
| Lack of inpatient bed | 1 (0.4%) | 0 |
| No identified barrier | 168 (70.0%) | 217 (90.4%) |

**Figure S1. Cumulative frequency of suicide cases**
